# Effect of icaritin on autophagy-related protein expression in TDP-43-transfected SH-SY5Y cells

**DOI:** 10.7717/peerj.13703

**Published:** 2022-07-04

**Authors:** Yanjun Zhou, Nanqu Huang, Yuanyuan Li, Zhisheng Ba, Yong Luo

**Affiliations:** 1 Department of Neurology, Wuhan No. 1 Hospital, Tongji Medical College, Huazhong University of Science and Technology, Wuhan, Hubei, China; 2 Department of Neurology, Third Affiliated Hospital of Zunyi Medical University (The First People’s Hospital of Zunyi), Zunyi, Guizhou, China; 3 National Drug Clinical Trial Institution, Third Affiliated Hospital of Zunyi Medical University (The First People’s Hospital of Zunyi), Zunyi, Guizhou, China

**Keywords:** TDP-43, Icaritin, Autophagy, Alzheimer’s disease

## Abstract

**Objective:**

To study the protective effect and mechanism of icaritin (ICT) in a SH-SY5Y cells with virus-loaded TAR DNA-binding domain protein 43(TDP-43) by examining the effect of ICT on the expression of autophagy-related proteins in TDP-43-infected SH-SY5Y cells.

**Methods:**

A TDP-43-induced neuronal cell injury model was established by transfecting well-growing SH-SY5Y cells with virus loaded with the TDP-43 gene. The changes in cell viability were detected by the CCK-8 method. After successful transfection, the establishment of the model was verified by real-time quantitative PCR (qPCR) and Western blot methods. After the cells were subjected to drug intervention with ICT, the changes in the expression levels of TDP-43, cleaved Caspase-3, LC3 II/I, Beclin-1 and p62 were detected by Western blotting.

**Results:**

After ICT intervention, it was found that compared with that of the TDP-43 group, the cell viability of the TDP-43+ICT group increased, the expression level of TDP-43 decreased, and the expression levels of the apoptotic protein cleaved Caspase-3, autophagy protein Beclin-1, and LC3-II/I decreased, while the expression level of the autophagy protein p62 increased.

**Conclusion:**

ICT has a protective effect on the SH-SY5Y cell injury model transfected with TDP-43. This protective effect may be related to reducing the protein expression of TDP-43 and inhibiting autophagy.

## Introduction

TAR DNA-binding domain protein 43 (TDP-43) is a highly conserved rib nucleoprotein body consisting of 414 amino acids located on chromosome 1p36, which contains six exons. TDP-43 plays multiple roles in regulating gene expression at the transcriptional and translational levels (Guo & Shorter, 2017). When expressed in most human tissues and cell types, TDP-43 is mainly nonphosphorylated and mainly located in the nucleus, while under pathological conditions, TDP-43 translocated to cytoplasm (Nelson et al., 2019). Increasing evidence shows that the TDP-43 protein plays a role in the pathogenesis of diseases such as amyotrophic lateral sclerosis (ALS), front temporal lobar dementia (FTLD) and Alzheimer’s disease (AD), and an increasing number of experts have proposed TDP-43 proteinopathy (Gao et al., 2018; Jamerlan & An, 2020). At the same time, studies have confirmed that the regulation of autophagy can affect the deposition of Aβ plaques and Tau protein tangles in mice to improve the cognitive and behavioral deficits of mice (LaClair et al., 2016). Moreover, autophagy plays a role in clearing abnormal TDP-43 (Huang, Yan & Zhang, 2020; Kumar et al., 2021), and overexpression of TDP-43 in self-defense under pathological conditions can activate autophagy (Wang et al. 2015a; Wang et al. 2015b). Moreover, Caspase-3, the end product of apoptosis, is closely related to autophagy (Gu et al., 2021; Xue et al., 2020).

Icaritin (ICT) is a flavonoid compound and is a metabolite of icariin (ICA), both of which are the active ingredients in the extract of epimedium (Zhai et al., 2013). The molecular structure of ICT is smaller than that of ICA, and ICT more easily penetrates the blood–brain barrier. ICT contains the chemical structure of polyphenolic hydroxyl groups, which have the functions of scavenging free radicals, anti-neuritis, neuroprotection and delaying aging (Jin et al., 2019; Zhang et al., 2021). Many studies have confirmed that ICA has a preventive effect on dementia model rats, and its mechanism can improve the cognitive decline of mice by reducing Aβ protein deposition and inhibiting inflammation, thereby delaying the occurrence of dementia (Liu et al., 2019; Mi et al., 2018; Sheng et al., 2017). Previous research by this group has shown that ICT can reduce the neurotoxicity of Aβ and Tau proteins to protect nerve cells (Feng et al., 2019; Feng et al., 2017; Li et al., 2021). At the same time, ICT reduces the expression of TDP-43 protein by reducing mitochondrial damage and reducing oxidative stress, thereby protecting cells (Zhou et al., 2021).

Therefore, combined with the previous results of this research group, TDP-43-transfected SH-SY5Y cells were the research focus of this study and we used ICT for drug intervention in SH-SY5Y cells carrying the viral TDP-43 protein. Then, we initially explored whether ICT has a protective effect on nerve cell damage caused by TDP-43 transfection. At the same time, it was clear that it exerts a protective effect through the autophagy pathway, which provides a certain basis for the treatment of TDP-43 proteinopathy and drug development.

## Material and Methods

### Culture and treatment of cells

SH-SY5Y human neuroblastoma cells donated by the Collection Center of Wuhan University were inoculated into 25 cm^2^ culture flasks and cultured in a 5% CO_2_ incubator at 37 °C. The medium was replaced every 24–48 h and was composed of DMEM-F12, 15% fetal bovine serum, and 1% penicillin. When it grew to occupy 80–90% of the bottom of the bottle, the cells were passaged. The packaged lentivirus was transfected into cells, 200 µL of virus-containing medium was added to each well of a 96-well plate, and 1 ×10^4^ cells were inoculated on the plate at an MOI of 0–60. The cell morphology was observed after 12 h of culture and the best MOI was selected.

### Cell viability assay

The CCK-8 assay was used to detect cell viability. The cells were grown under good conditions, the cell concentration was diluted to 5 ×10^4^/mL with culture medium, and the suspension was seeded in a 96-well plate with 200 µL per well. After culturing for 1 day, the medium was changed, 10 µL of CCK-8 solution was added to each well, and the cells were incubated in a 37 °C incubator for 2 h. Then, a Synergy HTX microplate reader was used to measure the absorbance at 450 nm and calculate the cell viability.

### Real-time quantitative PCR and qPCR

First, RNA was extracted, 200 µL of TransZol Up was added to the successfully transfected cells, 40 µL of chloroform was added and shaken for 30 s after the precipitate disappeared, and the mixture was allowed to stand. Then, 500 µL of CB9 and 500 µL of WB9 were added to the tube and discarded by centrifugation. The supernatant was finally added to 50 µL of RNase-free water, placed in an RNase-free tube and centrifuged to extract RNA. At a constant temperature of 65 °C, the mixing solution consisting of the primers shown in Table 1 was mixed with the extracted RNA and RNase-free water for 5 min and then placed on ice for 2 min, after which the other reaction components shown in Table 2 were added. According to the instructions, the corresponding number of reagents was added, followed by the corresponding steps of predenaturation, denaturation, annealing, extension to control the temperature, and the reaction time was controlled to form a dissolution curve to collect signals.

**Table 1:** Sequences of carrier primers.

Name	Sequences
GAPDH forward primer	GGAGCGAGATCCCTCCAAAAT
GAPDH reverse primer	GGCTGTTGTCATACTTCTCATGG
qTDP43-F1	AGGTGGCTTTGGGAATCAGG
qTDP43-R1	CCCAACTGCTCTGTAGTGCT

**Notes.**

The carrier primers were designed by Sangon Biotech Corporation, Ltd. (Shanghai, China).

**Table 2:** Reagents and volume.

Component	Volume
Total RNA	50 ng–5 µg
Anchored Oligo (dT)_18_ primer (0.5 µg/µL)	1 µL
Or Random Primer (0.1 µg/1 µL)	1 µL
2x ES Reaction Mix	10 µL
EasyScript RT/RI Enzyme Mix	1 µL
gDNA Remover	1 µL
RNase-free water	Variable
Total volume	20 µL

### Western blot

The cells were placed on ice, RIPA lysis buffer was added, and the cells were lysed on ice for 30 min. After centrifugation, the supernatant was taken as the protein sample. Then, the BCA method was used to determine the protein concentration. A standard curve was drawn at 562 nm on the microplate reader, and the blank of the standard curve was used as a control to determine the protein content of the sample. The protein was placed in boiling water at 100 °C and heated for 5 min to denature the protein. The separation gel and concentrated gel were selected according to the molecular weight of the target protein. Each 12% SDS–PAGE gel was added to 25 µg of total protein for separation. The concentrated gel electrophoresis voltage was set to 90 V, and electrophoresis was performed for approximately 30 min. After the same level, the separation gel electrophoresis voltage was set to 120 V, electrophoresis was performed for approximately 90 min, the electrophoresis was stopped when the sample band reached the bottom layer of the separation gel, and then transmembrane the protein. The nitrocellulose (NC) membrane was sealed with 5% skimmed milk powder for 1 h and washed with TBST three times after the sealing was completed, each time for 10 min. The NC membrane was incubated with the primary antibody overnight. After washing the membrane, the NC membrane was incubated with HRP-anti-rabbit antibody for 2 h. A color developing agent was added to the film and exposed in a fully automatic chemiluminescence gel imager, and then protein detection was performed after exposure.

### Statistical analysis

All statistical analyses were performed with IBM SPSS Statistics 22.0. If the data met normality, the mean ± SEM was used. The difference between the means of more than two groups was analyzed by one-way analysis of variance (ANOVA). If there were two variables in the statistical plot, two-way ANOVA was performed. When ANOVA showed significant differences, pairwise comparisons of means were made by Bonferroni’s post-hoc t test with correction. A value of *P* < 0.05 was considered statistically significant.

**Figure 1:**
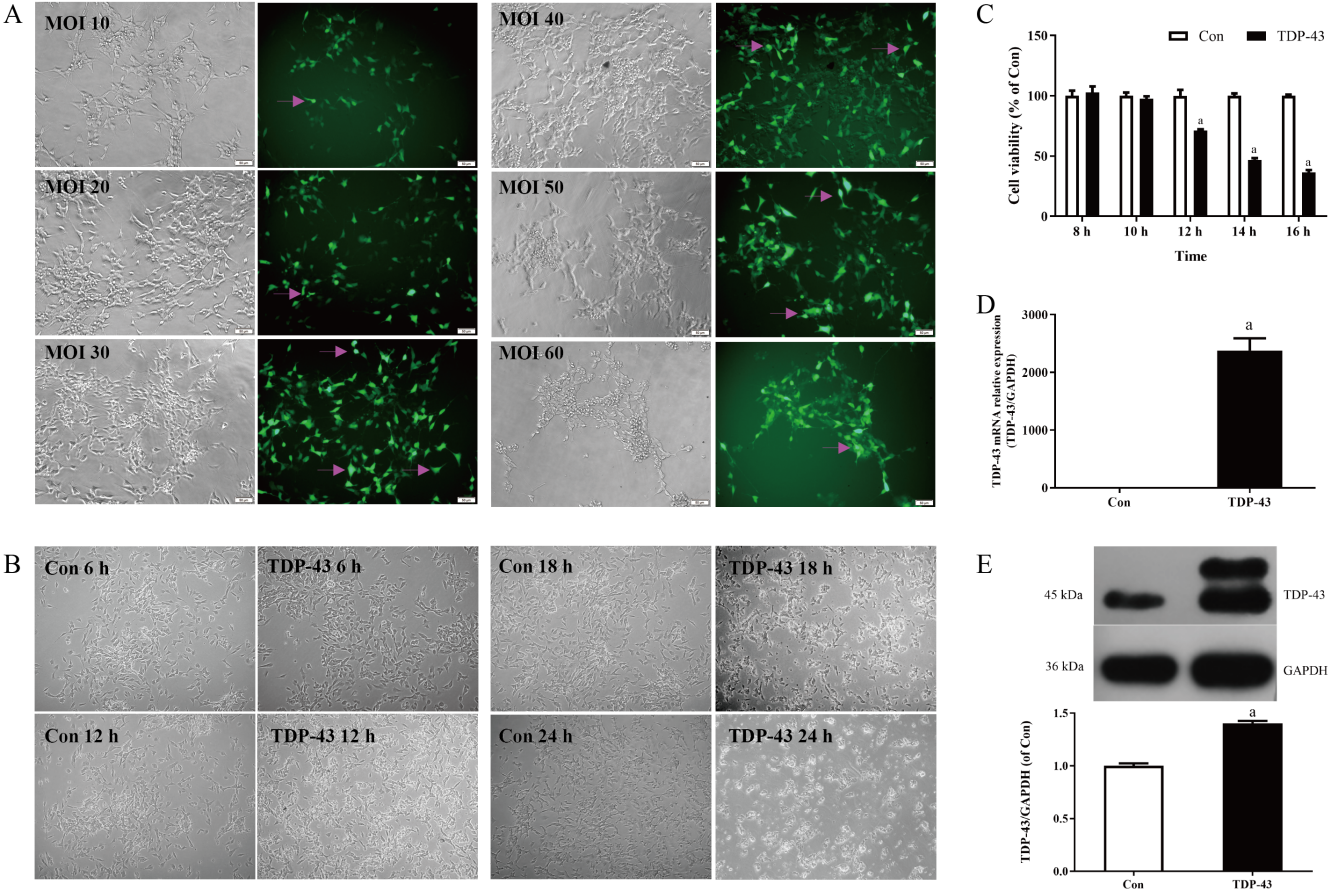
Establishment and verification of TDP-43-transfected SH-SY5Y cells. (A) Morphology of SH-SY5Y cells transfected with different concentrations of MOI virus under a microscope at 200×, magenta arrows indicate fluorescence. (B) Changes in SH-SY5Y cells at different infection times under a microscope at 100×. (C) Changes in cell viability 8, 10, 12, 14, and 16 h after TDP-43 transfection (^*a*^*P* < 0.05 *vs.* Con, *n* = 5). (D) mRNA expression of TDP-43 in SH-SY5Y cells transfected with *TDP-43* (^*a*^*P* < 0.05 *vs.* Con, *n* = 4). (E) Levels of TDP-43 in SH-SY5Y cells transfected with TDP-43. (^*a*^*P* < 0.05 *vs.* the Con, *n* = 3).

## Results

### Establishment and verification of the TDP-43-transfected SH-SY5Y cell model

Transfection was carried out with different concentrations of virus MOI. When the MOI was 30, the cell dendrites were clear, the growth state was good, and the transfection efficiency was high. When the MOI was 10 and 20, the cell state was good, but the transfection efficiency was low; when the MOI was 40, the transfection in the staining efficiency was good, but the cell status was less than when the MOI was 30, and the amount of cell death was greater than when the MOI was 30; the cell status was poor, the amount of cell death was greater and the transfection efficiency was low when the MOI was 50 and 60. Therefore, in this experiment MOI 30 was selected for transfection, as shown in Fig. 1A. At 18 h and 24 h after transfection, the cells in the TDP-43 group showed obvious shrinkage and a large amount of cell death; at 6 h after transfection, the cell dendrites were clear, and the growth status was good, without obvious cell death; at 12 h after transfection, the cell dendrites were clear, the growth state was acceptable, and there was a small amount of cell death, as shown in Fig. 1B. The effects of different transfection times at 8, 10, 12, 14, and 16 h on cell viability were measured. The results showed that after 12, 14, and 16 h of transfection, the viability of the TDP-43 group was lower than that of the Con group, and the viability of the cell model in the TDP-43 group decreased significantly after 12 h of transfection, suggesting the establishment of a cell injury model. Because TDP-43 has strong cytotoxicity, cell viability was significantly reduced after 14 and 16 h of transfection, and a large number of cells died. However, 12 h after transfection, the cells began to be damaged, and the cell condition was relatively good, so transfection for 12 h was chosen as the modeling condition, as shown in Fig. 1C. Based on the previous experimental results, the MOI was selected as 30, transfection lasted 12 h, and the model was established. At the same time, the expression of TDP-43 mRNA and protein in the model was verified. The expression of TDP-43 mRNA and protein was significantly higher than that in the Con group. All of the above results indicated successful modeling, which indicated that the virus had been transfected into the cell, and successful modeling was verified, as shown in Figs. 1D, 1E.

### The optimal concentration and duration of ICT and the effect of ICT on cell viability in the TDP-43 model

**Figure 2:**
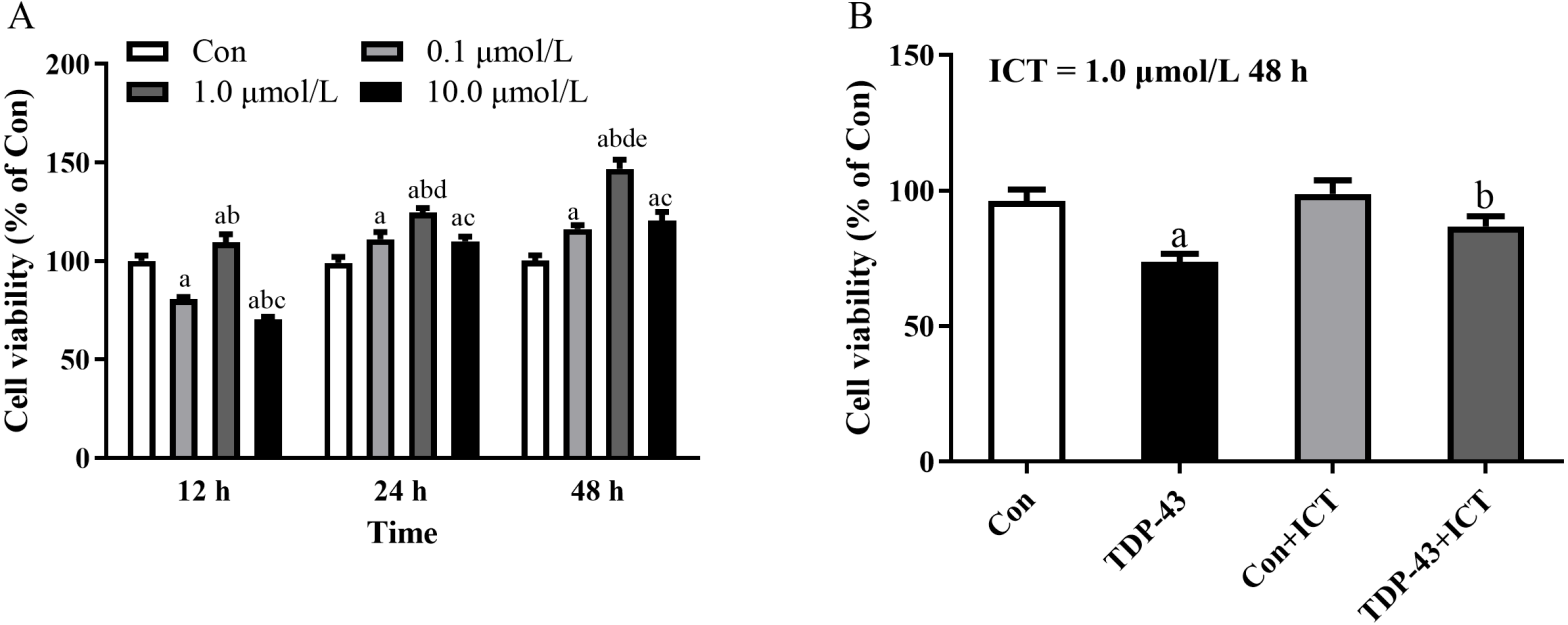
The optimal concentration and duration of ICT and the effect of ICT on cell viability in the TDP-43 model. (A) Changes in cell viability at different concentrations of ICT and different times (a means compared with 0.0 µmol/L ICT group, b means compared with 0.1 µmol/L ICT group, c means compared with 1.0 µmol/L ICT group; d means compared with 12 h, e means compared with 24 h compared with, *P* < 0.05, *n* = 5). (B) Changes in cell viability in the TDP-43-SH-SY5Y model after ICT intervention (^*a*^*P* < 0.05 *vs.* the Con, ^*b*^*P* < 0.05 *vs.* the TDP-43, *n* = 3).

After the cell model was established, ICT was added at different concentrations, including 0.0, 0.1, 1.0, and 10.0 µmol/L, to the TDP-43 group, and ICT was added for 12 h, 24 h, and 48 h to detect the cell viability of each group. The results showed that the cell viability of each group was the highest when the ICT concentration was 1.0 µmol/L and the incubation time was 48 h, as shown in Fig. 2A. Moreover, the results showed that the cell viability of the TDP-43 group was significantly reduced compared with that of the Con group. The cell viability of the 43+ICT group was higher than that of the TDP-43 group, as shown in Fig. 2B.

### Changes in the protein expression of autophagy- and TDP-43 and apoptosis-related proteins after ICT intervention

**Figure 3:**
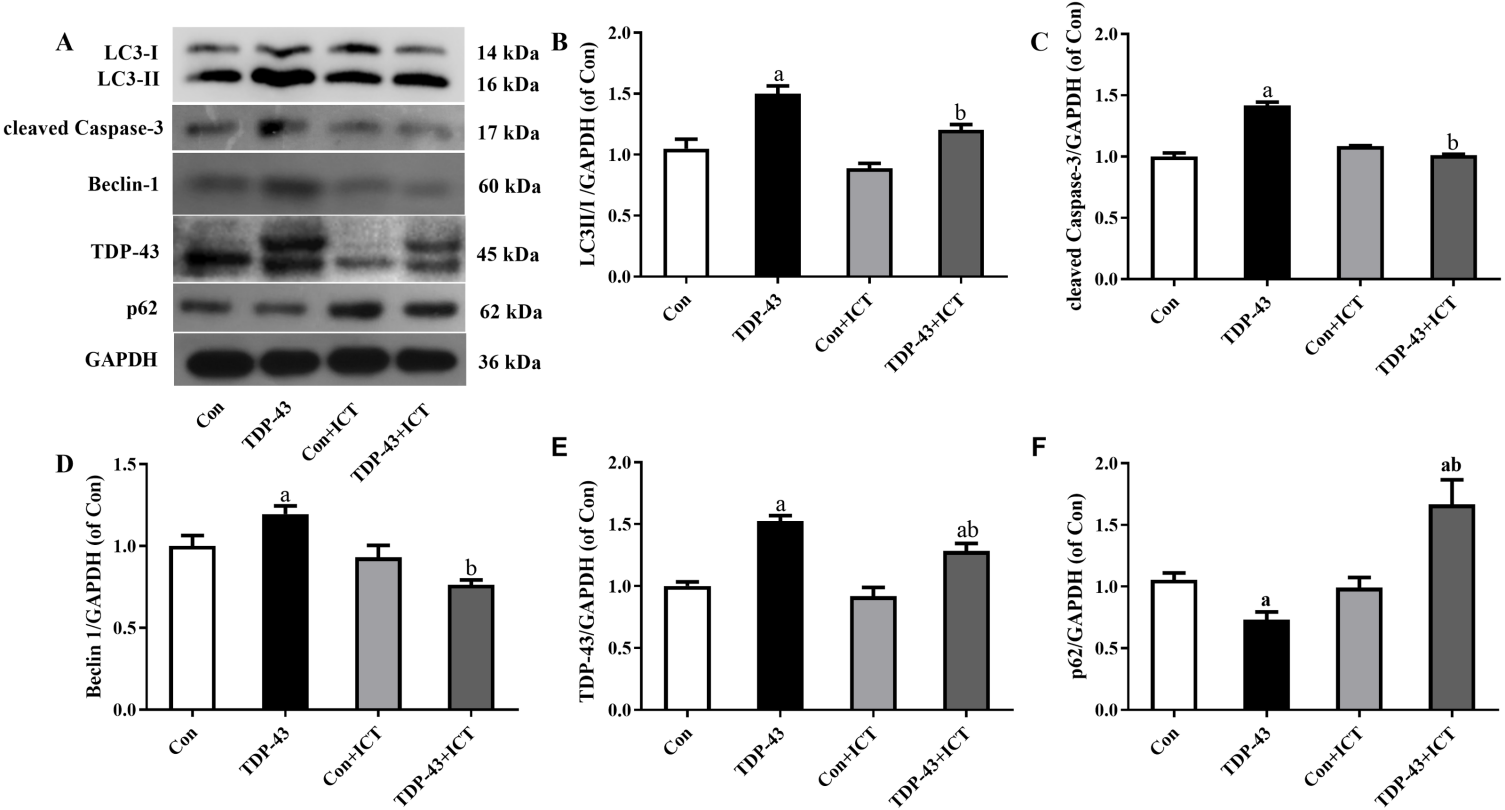
Levels of LC3 II/I, cleaved Caspase-3, Beclin-1, TDP-43, and p62 in TDP-43-transfected SH-SY5Y cells after ICT treatment. (A) Representative bands, (B) LC3 II/I, (C) cleaved Caspase-3, (D) Beclin-1, (E) TDP-43, (F) p62 (^*a*^*P* < 0.05 *vs.* the Con, ^*b*^*P* < 0.05 *vs.* the TDP-43, *n* = 3).

The expression levels of LC3-II/I, cleaved Caspase-3, Beclin 1 and TDP-43 in the TDP-43 group were significantly higher than those in the Con group, and the expression levels of LC3-II/I, cleaved Caspase-3, Beclin 1 and TDP-43 in the TDP-43+ICT group after 48 h of ICT treatment were clearly decreased than those in the TDP-43 group. At the same time, the expression of p62 in the TDP-43 group was lower than that in the Con group, and the expression of TDP-43+ICT after 48 h of ICT was significantly higher than that in the TDP-43 group, as shown in Fig. 3.

## Discussion

TDP-43 is a member of the nuclear heterogeneous ribonucleoprotein family and includes two domains of RNA recognition motifs located on both sides of the N-terminal and C-terminal regions. The C-terminus affects the binding of heterogeneous and homologous proteins, while the N-terminus is mainly involved in the formation of homodimers (Ayala et al., 2008; Kuo et al., 2009). TDP-43 regulates RNA splicing, microRNA biological production, and RNA transcription (Sephton et al., 2012). TDP-43 is a DNA/RNA binding protein mainly located in the nucleus under physiological conditions. However, the mislocalization of TDP-43 to the cytoplasm or the phosphorylation of TDP-43 can lead to the occurrence of diseases (Feneberg et al., 2018). At present, studies have shown that the cognitive function of patients with TDP-43 is significantly lower than that of patients without TDP-43 (Nelson et al., 2019). At the same time, retrospective studies have elaborated that TDP-43 is related to the incidence of hippocampal atrophy (Josephs et al., 2017). We found that TDP-43 accumulates in nerve cells of nearly all cases of amyotrophic lateral sclerosis and in the majority of Tau-negative FTLD (Cook et al., 2020; Feneberg et al., 2018).

Autophagy is a regulated self-degradation process in cells that is highly conserved in many species, such as mammals and yeast. Autophagy avoids the accumulation of misfolded or aggregated proteins, which is essential to maintain the normal function of the central nervous system. The autophagy process includes the process of initiating autophagy after receiving the signal, forming autophagosomes, autophagolysosomes formed by the combination of lysosomes and autophagosomes, degrading autophagolysosomes into small molecules and reusing decomposition products (Glick, Barth & Macleod, 2010). The initiation of autophagy involves the PI3K protein kinase complex and other substances. Beclin-1 is a subunit of the class III PI3K complex. As one of the indispensable proteins for the formation of autophagosomes, Beclin-1 is produced by autophagosomes. In this process, the autophagy precursor binds to Beclin1 protein to initiate the process. The formation of autophagosomes requires playing a role in the microtubule-associated protein light chain 3/autophagy-associated 8 homolog protein binding system. There are many homologs of Atg8 in mammalian cells, and LC3 is one of them. Atg4 has endoproteinase activity, and it cleaves the LC3/Atg8 protein binding system at the carboxyl end to produce LC3-I. After LC3-I undergoes a ubiquitin-like reaction, it is coupled with phosphatidylethanolamine to form LC3-II. LC3-II/I is a recognized marker for the evaluation and determination of autophagy (Ghavami et al., 2014; Yu, Chen & Tooze, 2018). As a kind of adaptor protein, p62 can be connected to the marked misfolded or aggregated protein through ubiquitin, and the other end is connected to the LC3 protein, which mediates the autophagic lysosome formation process. Finally, the adaptor protein is degraded along with the misfolded or aggregated protein. Studies have found that the level of p62 protein is negatively correlated with autophagy activity within a certain range. As p62 is a substrate for autophagy, an increase in its protein level can indicate a decrease in autophagy activity (Xiong et al., 2013). Autophagy and apoptosis are the basic physiological processes that maintain cell homeostasis, and research has shown that autophagy and apoptosis have connections (Li et al., 2020a). Cysteinyl aspartate specific proteinase (Caspase) is used for cell apoptosis. The main transmitters of death can be divided into promoters (such as Caspase-8 and Caspase-9) and effectors (such as Caspase-3 and Caspase-7) (Ghavami et al., 2014). Among them, studies have found that TDP-43 can be cleaved by the effector Caspase-3 into fragments of relatively small molecular weight (Igaz et al., 2009). Studies have shown that the human autophagy gene Beclin 1 has two Caspase cleavage sites, and Caspase-3, which is the final effector enzyme of the Caspase cascade reaction, is one of them. At the same time, a study showed that Caspase-3 can control cell autophagy by cleaving the autophagy regulator unc-51 like autophagy activating kinase 1 (ULK1) to control the occurrence of diseases (Abraham et al., 2016). There is evidence that TDP-43 regulates autophagy. In the case of TDP-43-related protein diseases, the loss of cell function caused by TDP-43 aggregates can further increase cellular stress by preventing the autophagy process, thereby delaying the onset or development of neurodegenerative diseases (Caccamo et al., 2015; Perera et al., 2021). Moreover, studies have found that the exogenous overexpression of TDP-43 continuously activates autophagy and promotes apoptosis in the case of insufficient nutrition (Lin et al., 2017).

Previous research by our group showed that ICT can inhibit β-amyloid precursor protein cleavage enzyme 1 (BACE 1) to block the production of amyloid precursor protein (APP) to reduce the secretion of Aβ (Feng et al., 2019). We found that ICT can decrease the levels of GSK-3β and p-Tau in model cells (Li et al., 2021). The results of our team suggested that ICT might have potential therapeutic benefits by reducing BACE-1 levels and Aβ_1−42_ contents (Li et al., 2020b). Our group also suggested that ICT has a protective effect on TDP-induced SH-SY5Y cell injury by reducing TDP-43 expression, mitigating mitochondrial injury and ameliorating oxidative stress (Zhou et al., 2021).

Moreover, studies have shown that ICA can act on diseases through autophagy and other pathways (Chen et al., 2019; Jiang et al., 2019). Therefore, we propose that ICT, which is a metabolite of ICA, may act through the autophagy pathway and be used as an effective drug for the treatment of TDP-43 proteinopathy.

Therefore, in this study, after transfecting SH-SY5Y cells with a virus carrying TDP-43, the cell morphology shrank, axons and dendrites were shortened or even disappeared, and cell death occurred. At the same time, the mRNA and protein expression of TDP-43 was measured. A significant increase in cell viability measured by CCK-8 decreased, and the cells in this state could better simulate the damage caused by TDP-43 in SH-SY5Y cells. Therefore, this experiment selected a virus carrying TDP-43 to transfect SH-SY5Y cells. Cell-based methods were used to establish cell models for research. Based on related literature and preliminary experiments, it was determined that the duration of virus transfection has different effects on the cells. Therefore, in this experiment, the cell viability of SH-SY5Y cell models after different transfection times was measured, and it was found that the cell viability was 12 h after transfection. The cell morphology changes were viewed under the microscope. At the same time, the cell viability was restored after ICT was added, and the cell morphology was partially restored to normal. Considering that TDP-43 has a greater impact on cytotoxicity, in this experiment the virus that carried TDP-43 was selected. SH-SY5Y cells were stained for 12 h as the experimental conditions for establishing cell models.

After the model was successfully established, according to the experimental results of this research group and previous experiments, the cell viability was the best when the SH-SY5Y cell model transfected with TDP-43 was treated with ICT at a concentration of 1 µmol/L for 48 h. After ICT drug intervention for 48 h, the cell morphology and growth status were significantly improved compared to before the drug was added. CCK8 assays showed that SH-SY5Y cell viability increased significantly after TDP-43 transfection compared with before the drug was added. Western blot results showed that after drug intervention, the expression of TDP-43 in the TDP-43+ICT group was lower than that in the TDP-43 group. At the same time, the expression of the apoptosis protein cleaved Caspase-3, autophagy protein Beclin-1, and LC3-II/I decreased after adding ICT, and the expression of the autophagy protein p62 increased, suggesting that ICT may affect the protein expression of cleaved Caspase-3, Beclin-1, LC3-II/I and p62, and it also has a protective effect on the autophagy pathway in the injury model of TDP-43-transfected SH-SY5Y cells.

In summary, this study used TDP-43-carrying virus to transfect SH-SY5Y cells and establish a TDP-43 transfection cell injury model. After ICT intervention, it was found that ICT reduced the autophagy pathway Beclin-1 and LC3-II/I protein expression by reducing cleaved Caspase-3 and increasing the expression level of p62 protein, thereby protecting the TDP-43-induced cell injury model. However, the current research on ICT is still in the preliminary stage, and the specific mechanism of its protective effect on the TDP-43-induced cell injury model is still unclear. This study explores the protective effect of ICT at the cellular level. In a follow-up study, our group will further verify its protective effect at the *in vivo* level and explore its protective mechanism to provide new ideas for the development of novel drugs for the treatment of TDP-43 proteinopathy.

## Conclusion

ICT has a protective impact on the SH-SY5Y cell injury model transfected with TDP-43. This protective effect may be relevant to regulating TDP-43, inhibiting the expression of cleaved Caspase-3, Beclin-1 and LC3-II/I and promoting the expression of p62 protein.

## Supplemental Information

10.7717/peerj.13703/supp-1 Supplemental Information 1 Full-length uncropped blots

10.7717/peerj.13703/supp-2 Supplemental Information 2 Raw numerical data for cell assays and qRT-PCR (Figs. 1–3)

## Additional Information and Declarations

### Data Availability

The following information was supplied regarding data availability: The raw measurements are available in the Supplemental Files.
