# Supplementary material for: Effect of icaritin on autophagy-related protein expression in TDP-43-transfected SH-SY5Y cells
Source: PeerJ. 2022 Jul 4;10:e13703. doi: 10.7717/peerj.13703 (PMC9261921; doi:10.7717/peerj.13703)
Supplement: Supplemental Information 1 [file peerj-10-13703-s001.docx]

**Full-length uncropped blots**

**Materials and methods of Western blot assay**

The cells were placed on ice, adding RIPA lysis buffer, lyse on ice for 30 minutes, and centrifuged in a 4℃ at 12000 rpm for 15 minutes, and then take the supernatant from the centrifuge tube as the protein sample. Then using the BCA method to determine the protein concentration, it drew a standard curve at 562 nm on the microplate reader, and used the blank of the standard curve as a control to find out the protein content of the sample. Placing the protein in boiling water at 100°C and heat it for 5 min to denature the protein. Selecting separation gel and concentrated gel according to the molecular weight of the target protein. Each 12% SDS-PAGE gel was add 25 µg of total protein for separation. Setting the concentrated gel electrophoresis voltage to 90 V and perform electrophoresis for about 30 minutes. After the same level, set the separation gel electrophoresis voltage to 120 V, electrophoresis for about 90 minutes, stop the electrophoresis when the sample band reaches the bottom layer of the separation gel, and transfer the protein to the nitrocellulose (NC) membrane. The NC membrane was sealed with 5% skimmed milk powder for 1 hour, and washed with TBST 3 times after the sealing was completed, each time for 10 min. Incubate the NC membrane with antibody one overnight. After washing the membrane, incubate the NC membrane with HRP-anti-rabbit antibody for 2 h. Add a color developing agent to the film and expose it in a fully automatic chemiluminescence gel imager, and then perform protein detection after exposure.

**Figure 1** Establishment and verification of TDP-43 transfected SH-SY5Y cell model.
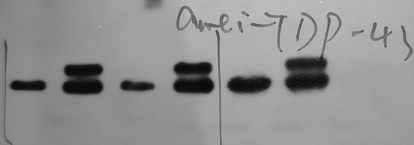


TDP-43-From left to right are Control, TDP-43, Control, TDP-43, Control, TDP-43 group.


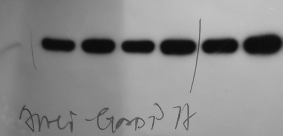


GAPDH-From left to right are Control, TDP-43, Control, TDP-43, Control, TDP-43 group.

**Figure 3** Levels of LC3 II/I, Caspase-3, Beclin-1, TDP-43, and p62 in TDP-43 transfected SH-SY5Y cells after ICT treatment.


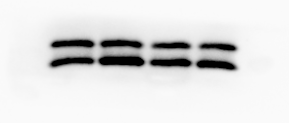


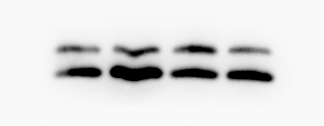


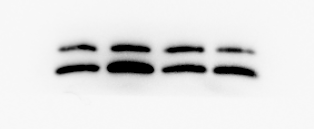


LC3 II/I-From left to right are Control, TDP-43, Control+ICT, TDP-43+ICT.


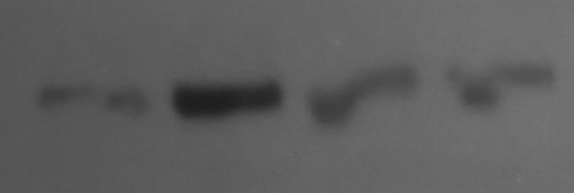


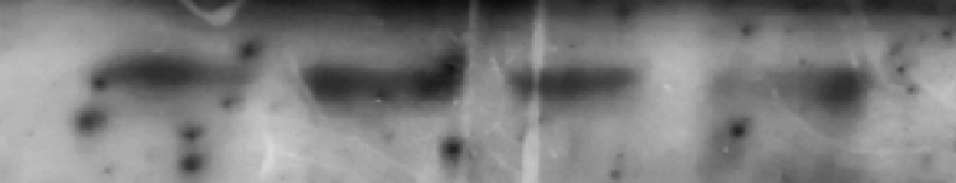


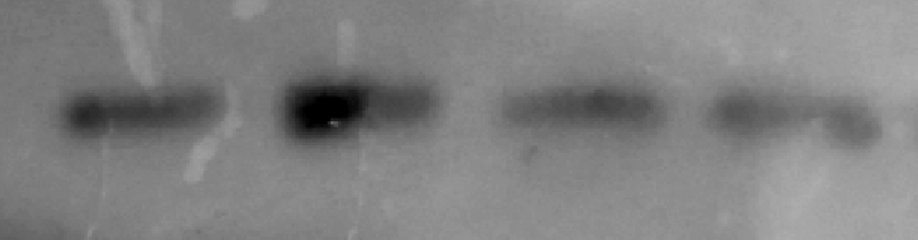


cleaved Caspase-3-From left to right are Control, TDP-43, Control+ICT, TDP-43+ICT.


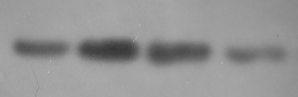


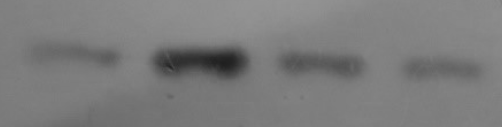


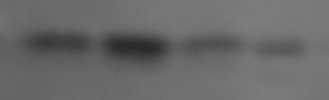


Beclin-1-From left to right are Control, TDP-43, Control+ICT, TDP-43+ICT.


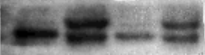


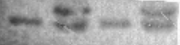


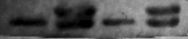


TDP-43-From left to right are Control, TDP-43, Control+ICT, TDP-43+ICT.


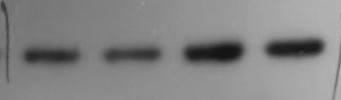


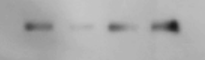


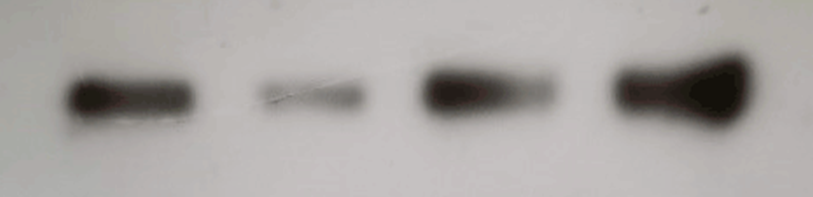


p-62-From left to right are Control, TDP-43, Control+ICT, TDP-43+ICT.


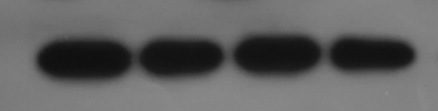


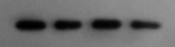


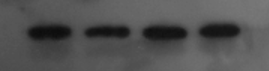


GAPDH-From left to right are Control, TDP-43, Control+ICT, TDP-43+ICT.
